# {2-[(Dimethyl­amino)­meth­yl]phen­yl}bis­(4-methyl­phen­yl)bis­muthane

**DOI:** 10.1107/S1600536810043655

**Published:** 2010-12-04

**Authors:** Masatoshi Kawahata, Shuji Yasuike, Izumi Kinebuchi, Kentaro Yamaguchi, Jyoji Kurita

**Affiliations:** a Faculty of Pharmaceutical Sciences at Kagawa Campus, Tokushima Bunri University, Shido, Sanuki 769-2193, Japan; b Faculty of Pharmaceutical Sciences, Hokuriku University, Kanagawa-machi, Kanazawa 920-1181, Japan

## Abstract

The title compound, [Bi(C_7_H_7_)_2_(C_9_H_12_N)], was obtained by treating chlorodi(*p*-tol­yl)bis­muthane with *o*-lithio-*N*,*N*-dimethyl­benzyl­amine. An intra­molecular Bi⋯N nonbonding inter­action is observed in the distorted trigonal triaryl­bis­muth coordination of the title compound.

## Related literature

For a review of the applications and structural chemistry of organobismuth compounds, see: Matano & Ikegami (2001 ▶); Silvestru *et al. *(1999 ▶). For related structural reports, see: Suzuki *et al.* (1993 ▶); Tokunaga *et al.* (2000*a* ▶,*b* ▶); Okajima *et al.* (2002 ▶).
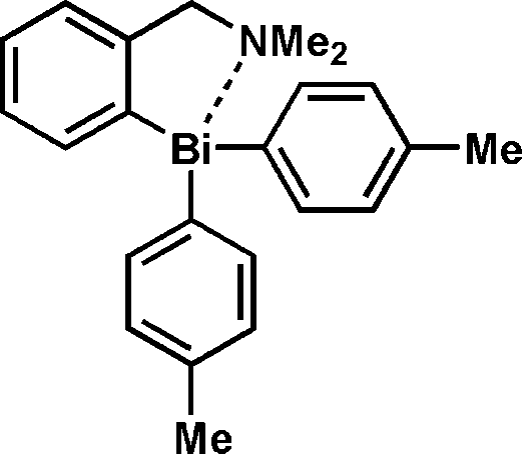


## Experimental

### 

#### Crystal data

[Bi(C_7_H_7_)_2_(C_9_H_12_N)]
*M* *_r_* = 525.43
Monoclinic, 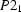

*a* = 6.0991 (12) Å
*b* = 19.630 (4) Å
*c* = 8.3699 (16) Å
β = 93.073 (2)°
*V* = 1000.6 (3) Å^3^
*Z* = 2
Mo *K*α radiation
μ = 8.81 mm^−1^
*T* = 100 K
0.20 × 0.08 × 0.01 mm

#### Data collection

Bruker APEXII CCD area-detector diffractometer
Absorption correction: multi-scan (*SADABS*; Sheldrick, 1996 ▶) *T*_min_ = 0.272, *T*_max_ = 0.917
4908 measured reflections
3626 independent reflections
3410 reflections with *I* > 2σ(*I*)
*R*_int_ = 0.016

#### Refinement

*R*[*F* ^2^ > 2σ(*F* ^2^)] = 0.019
*wR*(*F* ^2^) = 0.037
*S* = 1.00
3626 reflections
231 parameters
1 restraint
H-atom parameters constrained
Δρ_max_ = 0.86 e Å^−3^
Δρ_min_ = −0.59 e Å^−3^
Absolute structure: Flack (1983 ▶), with 1544 Friedel pairs
Flack parameter: 0.412 (7)

### 

Data collection: *APEX2* (Bruker, 2005 ▶); cell refinement: *SAINT* (Bruker, 2005 ▶); data reduction: *SAINT*; program(s) used to solve structure: *SHELXS97* (Sheldrick, 2008 ▶); program(s) used to refine structure: *SHELXL97* (Sheldrick, 2008 ▶); molecular graphics: *ORTEP-3 for Windows* (Farrugia, 1997 ▶); software used to prepare material for publication: *WinGX* (Farrugia, 1999 ▶).

## Supplementary Material

Crystal structure: contains datablocks I, global. DOI: 10.1107/S1600536810043655/si2298sup1.cif

Structure factors: contains datablocks I. DOI: 10.1107/S1600536810043655/si2298Isup2.hkl

Additional supplementary materials:  crystallographic information; 3D view; checkCIF report

## Figures and Tables

**Table:** 

C1—Bi1	2.291 (5)
C8—Bi1	2.265 (5)
C15—Bi1	2.267 (5)
N1—Bi1	2.902 (4)

**Table:** 

C1—Bi1—C8	96.07 (16)
C1—Bi1—C15	90.74 (16)
C1—Bi1—N1	157.55 (14)
C8—Bi1—C15	94.85 (17)
C8—Bi1—N1	81.26 (14)
C15—Bi1—N1	67.43 (13)

**Fig. 1.**
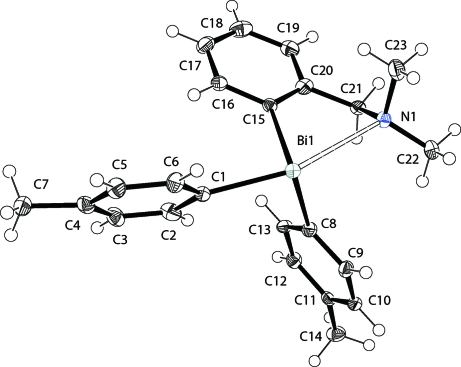
View of the title compound with the atom numbering scheme. The thermal ellipsoids are drawn at the 50% probability level. H atoms are shown as small spheres of arbitrary radii.

## supplementary crystallographic information

### Comment

Interest in the chemistry of organobisumuth(III) compounds has increased in recent years, due to the potential reagents and catalysts in organic synthesis as well as biological activity (Matano & Ikegami, 2001). Among these, the structural chemistry of bismuth compounds, including intramolecular interaction between bismuth and heteroatoms, has been widely reported in a review (Silvestru *et al.* 1999). On the other hand, we have recently reported the synthesis and structure of various organoantimony(III) compounds, such as 1-[8-(*N*,*N*-dimethylaminomethyl)naphthyl]bis(4-methylphenyl)stibane (Tokunaga *et al.*, 2000*a*), [2-(*N*,*N*-dimethylaminomethyl)phenyl]bis(4-methylphenyl)stibane (Tokunaga *et al.*, 2000*b*), and Sb(*S*)-[2-(*S*)-(*N*,*N*-dimethylaminomethyl)phenyl](1-naphthyl)(4-methylphenyl)stibane (Okajima *et al.*, 2002), bearing the CH_2_NMe_2_ moiety adjacent to the Sb atom as a pendant arm. X-ray crystal analyses of these compounds revealed the presence of intramolecular coordination between the Sb and N atoms. Here we report the synthesis and structure of the title compound, in which the central Sb atom of the [2-(*N*,*N*-dimethylaminomethyl)phenyl]bis(4-methylphenyl)stibane is replaced with Bi atom. The molecular structure and atom-numbering of the title compound are shown in Fig. 1. Selected geometric parameters are presented in Table 1. The analysis revealed that the Bi and three C (C1, C8, and C15) atoms exhibit a distorted trigonal-pyramidal arrangement with the Bi atom being far from the basal three-carbon plane (1.220 (3) Å). In addition, an intramolecular coordination between the Bi and N atoms is observed; the distance between the Bi and N atoms is 2.902 (4) Å, which corresponds to 74% of the sum of the van der Waals radii of both elements (3.94 Å) and accords with 131% of the covalent bond length (2.22 Å). It should be noted that the bond angle for C1—Bi1—N1 [157.55 (14)°] is significantly larger than those for C8—Bi1—N1 [81.26 (14)°] and C15—Bi1—N1 [67.43 (13)°], and the bond distance between Bi1 and C1 [2.291 (5) Å] is obviously longer than those for Bi1–C8 [2.265 (5) Å] and C15 [2.267 (5) Å]. The results imply that the central Bi atom is distorted equatorial vacant trigonal bipyramidal configuration with the N1 of the pendant arm and the C1 of the tolyl group being apical positions, similar to the geometry of chloro[2-(*N*,*N*-dimethylaminomethyl)phenyl](4-methylphenyl)bismuthane (Suzuki *et al.*, 1993). These results showed that the title compound is a hypervalent compound with 10-Bi-4 system, by analogy with the 10-Sb-4 system of the organoantimony compounds (Tokunaga *et al.*, 2000*a*,*b*; Okajima *et al.*, 2002).

### Experimental

The title compound was synthesized as follows: To a solution of *N*,*N*-dimethylbenzylamine (1.89 g, 14.0 mmol) in ether (25 ml) was added n-butyllithium (1.65 *M* in hexane, 10.2 ml, 16.8 mmol) at 273 K under an argon atmosphere, and the mixture was stirred for 24 h at room temperature. To this solution was added a suspension of chlorobis(4-methylphenyl)bismuthane [prepared by the redistribution reaction on the treatment of tris(4-methylphenyl)bismuthane (2.31 g, 4.8 mmol) and trichlorobismuthane (756 mg, 2.4 mmol) in ether (20 ml) at room temperature for 2 h] over 10 min at 273 K, and the mixture was stirred for 24 h at the same temperature. The mixture was quenched with water (100 ml) and diluted with CH_2_Cl_2_ (100 ml), and insoluble substances were removed by filtration. The organic layer was separated and the aqueous layer was extracted with CH_2_Cl_2_ (50 ml). The combined organic layer was washed with brine, dried and evaporated *in vacuo*. Purification of the residue by recrystallization from CH_3_CN gave 2-(*N,N*-dimethylaminomethyl)phenylbis(4-methylphenyl)bismuthane as colourless prisms (2.0 g, 53% yield; m.p. 372–374 K; ^1^H NMR (CDCl_3_): δ 1.98 (s, 6H), 2.30 (s, 6H), 3.40 (s, 2H), 7.14 (d, *J* = 7.3 Hz, 4H), 7.16 (m, 1H), 7.25 (m, 2H), 7.62 (d, *J* = 7.3 Hz, 4H), 7.80 (d, *J* = 6.9 Hz, 1H); ^13^C NMR (CDCl_3_): δ 21.5 (*q*), 44.5 (*q*), 67.1 (*t*), 127.2 (*d*), 129.3 (*d*), 129.6 (*d*), 130.8 (*d*), 136.5 (*s*), 137.7 (*d*), 139.6 (*d*), 145.1 (*s*), 155.9 (*s*), 158.5 (*s*); analysis calculated for C_23_H_26_BiN: C 52.58, H 4.99, N 2.67%; found: C 52.57, H 4.92, N 2.63%.

### Refinement

The H atoms were positioned geometrically (C—H = 0.93–0.96 Å) and were included in the refinement in the riding model approximation, with *U*_iso_(H) = *xU*_eq_(C, N), where *x* = 1.5 for methyl and *x* = 1.2 for all other H atoms. The crystal studied was a twin with the refined BASF ratio of 0.412 (7)/0.588 (7). The Flack parameter = 0.412 (7) was refined in the full matrix least-squares process using the TWIN/BASF option.

### Crystal data

**Table:**

[Bi(C_7_H_7_)_2_(C_9_H_12_N)]	*F*(000) = 508
*M**_r_* = 525.43	*D*_x_ = 1.744 Mg m^−^^3^
Monoclinic, *P*2_1_	Mo *K*α radiation, λ = 0.71073 Å
Hall symbol: P2yb	Cell parameters from 3137 reflections
*a* = 6.0991 (12) Å	θ = 2.4–26.9°
*b* = 19.630 (4) Å	µ = 8.81 mm^−^^1^
*c* = 8.3699 (16) Å	*T* = 100 K
β = 93.073 (2)°	Prismatic, colourless
*V* = 1000.6 (3) Å^3^	0.20 × 0.08 × 0.01 mm
*Z* = 2	

### Data collection

**Table:**

Bruker APEXII CCD area-detector diffractometer	3626 independent reflections
Radiation source: fine-focus sealed tube	3410 reflections with *I* > 2σ(*I*)
graphite	*R*_int_ = 0.016
φ and ω scans	θ_max_ = 27.0°, θ_min_ = 2.1°
Absorption correction: multi-scan (*SADABS*; Sheldrick, 1996)	*h* = −7→7
*T*_min_ = 0.272, *T*_max_ = 0.917	*k* = −22→24
4908 measured reflections	*l* = −10→5

### Refinement

**Table:**

Refinement on *F*^2^	Secondary atom site location: difference Fourier map
Least-squares matrix: full	Hydrogen site location: inferred from neighbouring sites
*R*[*F*^2^ > 2σ(*F*^2^)] = 0.019	H-atom parameters constrained
*wR*(*F*^2^) = 0.037	*w* = 1/[σ^2^(*F*_o_^2^) + (0.0116*P*)^2^] where *P* = (*F*_o_^2^ + 2*F*_c_^2^)/3
*S* = 1.00	(Δ/σ)_max_ = 0.001
3626 reflections	Δρ_max_ = 0.86 e Å^−^^3^
231 parameters	Δρ_min_ = −0.59 e Å^−^^3^
1 restraint	Absolute structure: Flack (1983), with 1544 Friedel pairs
Primary atom site location: structure-invariant direct methods	Flack parameter: 0.412 (7)

### Special details

**Table:**

Geometry. All s.u.'s (except the s.u. in the dihedral angle between two l.s. planes) are estimated using the full covariance matrix. The cell s.u.'s are taken into account individually in the estimation of s.u.'s in distances, angles and torsion angles; correlations between s.u.'s in cell parameters are only used when they are defined by crystal symmetry. An approximate (isotropic) treatment of cell s.u.'s is used for estimating s.u.'s involving l.s. planes.
Refinement. Refinement of *F*^2^ against ALL reflections. The weighted *R*-factor *wR* and goodness of fit *S* are based on *F*^2^, conventional *R*-factors *R* are based on *F*, with *F* set to zero for negative *F*^2^. The threshold expression of *F*^2^ > 2σ(*F*^2^) is used only for calculating *R*-factors(gt) *etc*. and is not relevant to the choice of reflections for refinement. *R*-factors based on *F*^2^ are statistically about twice as large as those based on *F*, and *R*-factors based on ALL data will be even larger.

### Fractional atomic coordinates and isotropic or equivalent isotropic displacement parameters (Å^2^)

**Table:**

	*x*	*y*	*z*	*U*_iso_*/*U*_eq_	
C1	0.6751 (8)	0.6439 (2)	0.4839 (6)	0.0163 (10)	
C2	0.8412 (8)	0.6254 (3)	0.5966 (6)	0.0230 (12)	
C3	0.8193 (8)	0.5693 (3)	0.6955 (6)	0.0232 (12)	
C4	0.6268 (8)	0.5298 (2)	0.6864 (6)	0.0204 (11)	
C5	0.4595 (8)	0.5495 (2)	0.5785 (6)	0.0216 (11)	
C6	0.4845 (8)	0.6055 (2)	0.4787 (6)	0.0199 (11)	
C7	0.6102 (9)	0.4657 (2)	0.7843 (6)	0.0261 (12)	
C8	0.9474 (8)	0.7916 (2)	0.4269 (6)	0.0169 (11)	
C9	0.8880 (8)	0.8577 (2)	0.4634 (6)	0.0181 (11)	
C10	1.0307 (8)	0.9002 (2)	0.5535 (6)	0.0185 (11)	
C11	1.2387 (8)	0.8775 (2)	0.6077 (6)	0.0158 (10)	
C12	1.2973 (8)	0.8115 (2)	0.5681 (6)	0.0190 (11)	
C13	1.1561 (8)	0.7696 (2)	0.4794 (6)	0.0176 (11)	
C14	1.3902 (8)	0.9223 (2)	0.7087 (6)	0.0240 (12)	
C15	0.9686 (7)	0.6605 (2)	0.1743 (5)	0.0134 (10)	
C16	0.9732 (8)	0.5895 (2)	0.2016 (6)	0.0197 (11)	
C17	1.1305 (9)	0.5485 (3)	0.1324 (6)	0.0248 (12)	
C18	1.2779 (9)	0.5775 (3)	0.0349 (7)	0.0286 (13)	
C19	1.2694 (8)	0.6474 (3)	0.0021 (6)	0.0220 (11)	
C20	1.1185 (18)	0.6885 (5)	0.0734 (12)	0.019 (2)	
C21	1.1160 (17)	0.7642 (5)	0.0378 (11)	0.0148 (19)	
C22	0.9016 (9)	0.8666 (2)	0.0186 (6)	0.0234 (12)	
C23	0.7809 (8)	0.7669 (2)	−0.1263 (6)	0.0248 (12)	
N1	0.8969 (6)	0.79253 (18)	0.0204 (5)	0.0154 (9)	
Bi1	0.70642 (2)	0.72364 (2)	0.286635 (16)	0.01465 (4)	
H2	0.9692	0.6512	0.6057	0.028*	
H3	0.9333	0.5577	0.7687	0.028*	
H5	0.3285	0.5251	0.5724	0.026*	
H6	0.3698	0.6174	0.4065	0.024*	
H7A	0.6739	0.4284	0.7290	0.039*	
H7B	0.4586	0.4560	0.8001	0.039*	
H7C	0.6874	0.4719	0.8863	0.039*	
H9	0.7511	0.8741	0.4273	0.022*	
H10	0.9866	0.9442	0.5778	0.022*	
H12	1.4351	0.7952	0.6022	0.023*	
H13	1.2011	0.7258	0.4542	0.021*	
H14A	1.3614	0.9164	0.8195	0.036*	
H14B	1.3662	0.9691	0.6790	0.036*	
H14C	1.5398	0.9102	0.6922	0.036*	
H16	0.8711	0.5698	0.2661	0.024*	
H17	1.1347	0.5019	0.1524	0.030*	
H18	1.3842	0.5506	−0.0097	0.034*	
H19	1.3657	0.6663	−0.0680	0.026*	
H21A	1.1975	0.7878	0.1237	0.018*	
H21B	1.1904	0.7721	−0.0600	0.018*	
H22A	0.9813	0.8819	−0.0705	0.035*	
H22B	0.9724	0.8829	0.1163	0.035*	
H22C	0.7541	0.8837	0.0086	0.035*	
H23A	0.8557	0.7820	−0.2180	0.037*	
H23B	0.6332	0.7840	−0.1326	0.037*	
H23C	0.7782	0.7180	−0.1241	0.037*	

### Atomic displacement parameters (Å^2^)

**Table:**

	*U*^11^	*U*^22^	*U*^33^	*U*^12^	*U*^13^	*U*^23^
C1	0.019 (3)	0.015 (2)	0.015 (3)	0.001 (2)	0.003 (2)	0.000 (2)
C2	0.019 (3)	0.030 (3)	0.019 (3)	−0.007 (2)	0.002 (2)	−0.002 (2)
C3	0.024 (3)	0.028 (3)	0.018 (3)	0.005 (2)	−0.002 (2)	0.004 (2)
C4	0.021 (3)	0.022 (3)	0.018 (3)	0.004 (2)	0.007 (2)	0.001 (2)
C5	0.018 (3)	0.021 (3)	0.027 (3)	−0.006 (2)	0.007 (2)	0.001 (2)
C6	0.017 (3)	0.019 (3)	0.024 (3)	0.004 (2)	−0.001 (2)	0.002 (2)
C7	0.034 (3)	0.018 (3)	0.026 (3)	0.002 (2)	0.009 (3)	0.002 (2)
C8	0.020 (3)	0.014 (2)	0.017 (3)	−0.0034 (19)	0.004 (2)	−0.0005 (19)
C9	0.020 (3)	0.015 (2)	0.019 (3)	0.002 (2)	−0.001 (2)	0.001 (2)
C10	0.027 (3)	0.014 (2)	0.016 (3)	0.001 (2)	0.007 (2)	0.000 (2)
C11	0.020 (3)	0.016 (2)	0.011 (3)	−0.005 (2)	0.002 (2)	0.0015 (19)
C12	0.017 (3)	0.021 (3)	0.019 (3)	0.003 (2)	−0.001 (2)	0.002 (2)
C13	0.022 (3)	0.015 (2)	0.016 (3)	0.000 (2)	0.004 (2)	−0.0033 (19)
C14	0.025 (3)	0.023 (3)	0.024 (3)	−0.005 (2)	0.004 (2)	−0.006 (2)
C15	0.013 (2)	0.015 (2)	0.013 (3)	0.0007 (19)	0.001 (2)	−0.0001 (19)
C16	0.026 (3)	0.017 (3)	0.015 (3)	−0.004 (2)	−0.004 (2)	−0.002 (2)
C17	0.031 (3)	0.017 (3)	0.026 (3)	0.003 (2)	−0.005 (3)	−0.003 (2)
C18	0.025 (3)	0.031 (3)	0.030 (3)	0.006 (2)	0.001 (3)	−0.011 (2)
C19	0.021 (3)	0.026 (3)	0.019 (3)	−0.006 (2)	0.002 (2)	−0.002 (2)
C20	0.017 (4)	0.020 (4)	0.020 (5)	0.000 (3)	−0.012 (3)	−0.004 (3)
C21	0.013 (4)	0.017 (4)	0.015 (4)	0.000 (3)	0.007 (3)	−0.001 (3)
C22	0.032 (3)	0.019 (3)	0.019 (3)	−0.001 (2)	0.004 (2)	0.006 (2)
C23	0.027 (3)	0.025 (3)	0.022 (3)	0.004 (2)	0.001 (2)	0.006 (2)
N1	0.013 (2)	0.016 (2)	0.017 (2)	−0.0011 (16)	0.0037 (17)	0.0023 (16)
Bi1	0.01336 (7)	0.01541 (7)	0.01517 (7)	0.0000 (2)	0.00051 (5)	−0.0003 (2)

### Geometric parameters (Å, °)

**Table:**

C1—C6	1.384 (6)	C14—H14A	0.9600
C1—C2	1.394 (6)	C14—H14B	0.9600
C1—Bi1	2.291 (5)	C14—H14C	0.9600
C2—C3	1.389 (7)	C15—C20	1.391 (12)
C2—H2	0.9300	C15—C16	1.412 (6)
C3—C4	1.405 (7)	C15—Bi1	2.267 (5)
C3—H3	0.9300	C16—C17	1.401 (7)
C4—C5	1.381 (7)	C16—H16	0.9300
C4—C7	1.509 (7)	C17—C18	1.370 (7)
C5—C6	1.394 (7)	C17—H17	0.9300
C5—H5	0.9300	C18—C19	1.400 (7)
C6—H6	0.9300	C18—H18	0.9300
C7—H7A	0.9600	C19—C20	1.383 (11)
C7—H7B	0.9600	C19—H19	0.9300
C7—H7C	0.9600	C20—C21	1.515 (7)
C8—C9	1.386 (6)	C21—N1	1.448 (10)
C8—C13	1.393 (6)	C21—H21A	0.9700
C8—Bi1	2.265 (5)	C21—H21B	0.9700
C9—C10	1.397 (6)	C22—N1	1.454 (6)
C9—H9	0.9300	C22—H22A	0.9600
C10—C11	1.397 (6)	C22—H22B	0.9600
C10—H10	0.9300	C22—H22C	0.9600
C11—C12	1.388 (6)	C23—N1	1.473 (6)
C11—C14	1.503 (6)	C23—H23A	0.9600
C12—C13	1.379 (6)	C23—H23B	0.9600
C12—H12	0.9300	C23—H23C	0.9600
C13—H13	0.9300	N1—Bi1	2.902 (4)
			
C6—C1—C2	117.3 (4)	C20—C15—C16	118.7 (6)
C6—C1—Bi1	116.8 (3)	C20—C15—Bi1	122.5 (5)
C2—C1—Bi1	125.4 (4)	C16—C15—Bi1	118.8 (4)
C3—C2—C1	121.3 (5)	C17—C16—C15	120.5 (5)
C3—C2—H2	119.4	C17—C16—H16	119.7
C1—C2—H2	119.4	C15—C16—H16	119.7
C2—C3—C4	120.8 (5)	C18—C17—C16	119.6 (5)
C2—C3—H3	119.6	C18—C17—H17	120.2
C4—C3—H3	119.6	C16—C17—H17	120.2
C5—C4—C3	117.9 (5)	C17—C18—C19	120.3 (5)
C5—C4—C7	121.3 (5)	C17—C18—H18	119.8
C3—C4—C7	120.7 (4)	C19—C18—H18	119.8
C4—C5—C6	120.7 (5)	C20—C19—C18	120.4 (6)
C4—C5—H5	119.6	C20—C19—H19	119.8
C6—C5—H5	119.6	C18—C19—H19	119.8
C1—C6—C5	122.0 (4)	C19—C20—C15	120.4 (8)
C1—C6—H6	119.0	C19—C20—C21	119.1 (11)
C5—C6—H6	119.0	C15—C20—C21	120.5 (10)
C4—C7—H7A	109.5	N1—C21—C20	113.4 (10)
C4—C7—H7B	109.5	N1—C21—H21A	108.9
H7A—C7—H7B	109.5	C20—C21—H21A	108.9
C4—C7—H7C	109.5	N1—C21—H21B	108.9
H7A—C7—H7C	109.5	C20—C21—H21B	108.9
H7B—C7—H7C	109.5	H21A—C21—H21B	107.7
C9—C8—C13	117.8 (4)	N1—C22—H22A	109.5
C9—C8—Bi1	119.7 (3)	N1—C22—H22B	109.5
C13—C8—Bi1	122.5 (3)	H22A—C22—H22B	109.5
C8—C9—C10	121.0 (4)	N1—C22—H22C	109.5
C8—C9—H9	119.5	H22A—C22—H22C	109.5
C10—C9—H9	119.5	H22B—C22—H22C	109.5
C9—C10—C11	120.9 (4)	N1—C23—H23A	109.5
C9—C10—H10	119.6	N1—C23—H23B	109.5
C11—C10—H10	119.6	H23A—C23—H23B	109.5
C12—C11—C10	117.4 (4)	N1—C23—H23C	109.5
C12—C11—C14	121.5 (4)	H23A—C23—H23C	109.5
C10—C11—C14	121.0 (4)	H23B—C23—H23C	109.5
C13—C12—C11	121.6 (4)	C21—N1—C22	111.6 (5)
C13—C12—H12	119.2	C21—N1—C23	110.6 (5)
C11—C12—H12	119.2	C22—N1—C23	110.0 (4)
C12—C13—C8	121.3 (4)	C21—N1—Bi1	98.6 (4)
C12—C13—H13	119.4	C22—N1—Bi1	118.9 (3)
C8—C13—H13	119.4	C23—N1—Bi1	106.7 (3)
C11—C14—H14A	109.5	C1—Bi1—C8	96.07 (16)
C11—C14—H14B	109.5	C1—Bi1—C15	90.74 (16)
H14A—C14—H14B	109.5	C1—Bi1—N1	157.55 (14)
C11—C14—H14C	109.5	C8—Bi1—C15	94.85 (17)
H14A—C14—H14C	109.5	C8—Bi1—N1	81.26 (14)
H14B—C14—H14C	109.5	C15—Bi1—N1	67.43 (13)
